# Accelerated atherosclerosis in Anti-Neutrophil Cytoplasmic Antibody-Associated Vasculitis: case report

**DOI:** 10.47487/apcyccv.v5i1.331

**Published:** 2024-03-19

**Authors:** Natalia Andrea Uribe Ruíz, María Paulina Villa, Alberto Álzate Gutiérrez, Clara Inés Saldarriaga Giraldo, José Gregorio Thorrens Ríos

**Affiliations:** 1 Universidad de Antioquia, Medellín, Colombia. Universidad de Antioquia Universidad de Antioquia Medellín Colombia; 2 Universidad Pontificia Bolivariana, Medellín, Colombia. Universidad Pontificia Bolivariana Universidad Pontificia Bolivariana Medellín Colombia; 3 Clínica Cardio VID, Medellín, Colombia. Clínica Cardio VID Medellín Colombia

**Keywords:** Atherosclerosis, Anti-Neutrophil Cytoplasmic Antibody-Associated Vasculitis, Microscopic Polyangiitis, Myocardial Infarction

## Abstract

Accelerated atherosclerosis has been identified as a complication of multiple autoimmune diseases, among which Anti-Neutrophil Cytoplasmic Antibody-Associated Vasculitis stands out. We describe the case of a 60-year-old patient with a history of hypertension, diabetes mellitus, and chronic kidney disease of unknown etiology, who presented two acute coronary syndromes with only a six-month difference. Rapid progression of coronary involvement was evidenced, along with increased markers of inflammatory response, usual interstitial pneumonia on tomography, and positive anti-myeloperoxidase antibodies (anti-MPO), leading to the diagnosis of microscopic polyangiitis (MPA). In these cases, timely diagnostic suspicion is crucial, as early treatment significantly impacts the course and prognosis of the disease.

## Introduction

Vasculitis are a group of disorders characterized by inflammation of the blood vessels, endothelial injury, and tissue damage with clinical manifestations that vary according to the type of vessel affected. One of the rare conditions of vasculitis is involvement of the coronary arteries ^1^^,^^2^. We present the case of a woman with acute coronary syndrome, in whom vasculitis associated with anti-neutrophil cytoplasmic antibodies (ANCA) with renal, pulmonary, and coronary involvement secondary to accelerated atherosclerosis was documented. Dual antiplatelet therapy and high-intensity statins were prescribed, which she adhered to adequately during oupatient follow-up.

## Case report

**Figura 1:**
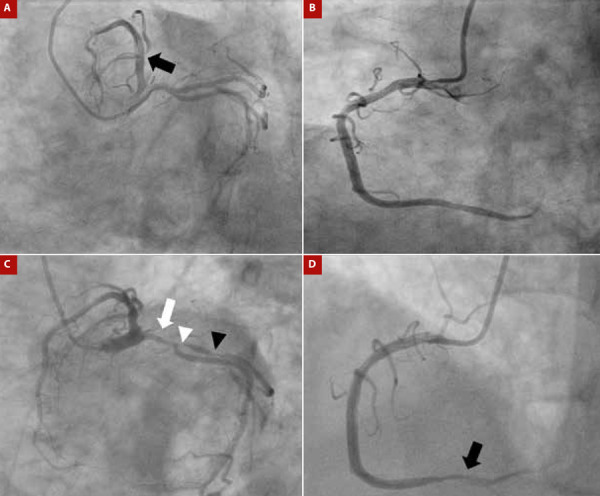
(A) Initial coronary angiography showing a non-significant lesion in the first diagonal artery (black arrow). (B) Initialcoronary angiography with dominant right coronary artery without lesions. (C) Second coronary angiography showing severe 70% stenosis of the proximal circumflex artery (white arrow) and severe lesions at the origin (white arrowhead) and proximal third (black arrowhead) of the obtuse marginal branch. D) Second coronary angiography showing severe 80% stenosis in the distal third of the right coronary artery (black arrowhead).

A 60-year-old woman with a history of hypertension, type 2 diabetes mellitus for 10 years, and stage 5 chronic kidney disease of unknown etiology on renal replacement therapy with peritoneal dialysis for 3 years presented with clinical features consistent with non-ST elevation acute myocardial infarction. Coronary angiography showed a non-significant lesion in the first diagonal artery (Figure 1A and 1B); echocardiography revealed no abnormalities in myocardial contractility and an ejection fraction of 60%. Physical examination revealed crackles in lung fields. An electrocardiogram showed evidence of repolarization disorder in the lateral wall, and further evaluation with high-sensitivity troponin and repeat coronary angiography revealed significant multivessel coronary artery disease (Figure 1C and 1D), with indication for revascularization.

**Figure 2:**
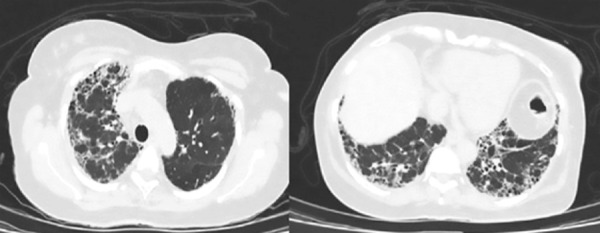
Contrast-enhanced chest tomography. Honeycombing involving apices and bases, predominantly in the subpleural and basal regions.

Given the findings of pulmonary auscultation and respiratory symptoms, a chest X-ray was performed (Figure 2), which revealed findings consistent with interstitial pneumonia. Metabolic studies showed a glycosylated hemoglobin level of 5.9% and a lipid profile with total cholesterol: 172 mg/dL; High-Density Lipoprotein (HDL): 32 mg/dL; triglycerides: 130 mg/dL; and Low-Density Lipoprotein (LDL): 114 mg/dL, which was above the target range, but did not explain the patient’s clinical course. Additionally, inflammatory markers were elevated, with a C-reactive protein level of 2.8 mg/dL (reference value [RV] <0.5 mg/dL) and erythrocyte sedimentation rate of 49 mm/h (RV <20 mm/h). Given the renal involvement, further differential diagnosis was pursued, revealing positivity for anti-myeloperoxidase (anti-MPO) antibodies via enzyme-linked immunosorbent assay (ELISA) at 41.9 IU (RV 0 to 20 IU).

A retrospective extramural search was also conducted, and a report of the renal biopsy performed at the time of diagnosis of kidney disease was found, which concluded in necrotizing glomerulonephritis with extracapillary proliferation in a chronic phase. This led to a diagnosis of microscopic polyangiitis (MPA) with renal, pulmonary (interstitial) involvement and accelerated coronary atherosclerosis. Finally, management included methylprednisolone 500 mg/day for 3 days, followed by prednisolone 30 mg/day with a clearance plan, and rituximab 1 g (day 0-14). It was deemed appropriate to wait until disease activity was controlled before proceeding with revascularization safely.

## Discussion

The clinical case illustrates a cardiac manifestation that, although rare, leads to significant morbidity and mortality. While the association between cardiovascular diseases and chronic inflammatory conditions is well described in patients with ANCA-associated vasculitis (AAV) ^1^, cardiac involvement poses a diagnostic challenge and requires a high clinical suspicion leading to timely intervention, as was the case in our patient.

Cardiovascular disease in autoimmune diseases can arise from various mechanisms, including accelerated atherosclerosis, valvular disease, systemic, myocardial and/or vascular inflammation, as well as myocardial ischemia secondary to microvascular, macrovascular, or direct coronary artery disease, which can lead to secondary myocardial fibrosis ^2^. Accelerated atherosclerosis is a complication of multiple systemic autoimmune diseases, particularly rheumatoid arthritis, systemic lupus erythematosus, psoriatic arthritis, and vasculitis ^3^^,^^4^.

Vasculitis causes vascular involvement through the direct leukocytes invasion into the walls of blood vessels, generating disproportionate inflammation that can lead to occlusion, stenosis, aneurysm, and/or rupture, or the development of premature atherogenesis ^3^. TNFα-mediated production of proinflammatory cytokines, mainly IL-1 and IL-6, is described, which leads to endothelial injury, blood vessel occlusion, and ischemia, resulting in organ damage. The necrosis of endothelial cells and detachment of the basement membrane not only provide a potential biomarker of disease activity but are also harmful. These structures derived from platelet activation and dead endothelial cell remnants form microparticles that have been associated with the formation of unstable plaques, known to mediate adverse cardiovascular events ^1^^,^^5^.

Atherosclerosis in AAV has been described in several studies reporting endothelial dysfunction, increased arterial stiffness measured by pulse wave velocity, and increased intima thickness ^6^. This phenomenon is characterized by abnormal accumulation of oxidized LDL in the arterial intima, leading to recruitment of monocytes, macrophages, phagocytosis, and activation and release of cytokines. Adaptive immunity plays an important role in pathophysiology as do neutrophil extracellular traps and the inflammasome. Recent evidence suggests that cholesterol crystals can induce activation of the innate immune system through assembly of the NLRP3 protein of the inflammasome (Nod-like receptor family pyrin domain-containing-3) resulting in production of IL-1β and IL-18. Inflammation stimulates secretion of metalloproteinases and apoptosis of smooth muscle cells, predisposing to destabilization of atherosclerotic plaques with potential for rupture and thrombosis ^3^.

AAV is associated with a significant risk of death, with an approximately 2.7-fold increase compared to the general population, with cardiac involvement being an important predictor of mortality ^3^. These antibodies can directly activate neutrophils, leading to the generation of reactive oxygen species and direct endothelial damage ^7^. In these patients, cardiovascular death is the most common cause (7.1%), followed by malignancy and infection; Terrier *et al.*, in a prospective cohort of 42 patients diagnosed with AAV in remission, documented that at 5 and 10 years, 9.5% and 26.8%, respectively, experienced a major cardiovascular outcome (myocardial infarction, stroke, or death) ^8^. Patients with vasculitis associated with anti-MPO antibodies have a higher risk of cardiovascular death compared to patients with vasculitis associated with anti-proteinase 3 (anti-PR3) antibodies, which may be due to more severe renal damage, typically occurring in patients with MPA. In fact, a possible chemotactic activity of oxidized LDL towards circulating T lymphocytes and monocytes has been described as part of the pathophysiology of accelerated atherosclerosis in AAV ^3^^-^^10^. Therefore, it has been suggested that early inhibition of anti-MPO antibodies may contribute to plaque stabilization and prevention of atherosclerotic lesion progression ^1^.

Major cardiovascular events occur 1.65 to 3 times more frequently in patients with AAV compared to the general population, and the risk appears to be higher in the first year after diagnosis. Age, systolic blood pressure, glomerular filtration rate, HDL levels, and the Birmingham Vasculitis Activity Score (BVAS) are described as independent factors, suggesting that systemic inflammation and traditional risk factors directly contribute to the increased risk in these patients ^2^^-^^11^. However, it is important to mention that atherosclerosis can develop even in patients in remission, as described by González-Suarez *et al.* in 23 patients with complete remission of AAV (39.6% with granulomatosis with polyangiitis), showing a high degree of subclinical atherosclerosis compared to the general population ^12^^,^^13^.

Other risk factors such as the presence of metabolic syndrome, impaired renal function, and persistent proteinuria are more common in patients with AAV; diabetes mellitus is 2 to 10 times more common and occurs in 7.2-29% of patients with AAV, while hypertension varies widely, affecting 9-83% of patients. Additionally, it has been described that lipid levels increase in the first 6 months after the diagnosis of AAV, possibly as a result of glucocorticoid therapy ^3^, leading to a higher prevalence of hypercholesterolemia with increases of up to 7.5 mg/dL in total cholesterol with minimal increases in steroid doses ^14^^,^^15^.

The above has led societies such as the European League Against Rheumatism to recommend active monitoring and modification of cardiovascular risk factors. This includes smoking cessation, weight control, increased physical activity, early identification, treatment and control of other comorbidities such as hypertension, diabetes, and hypercholesterolemia, while also emphasizing the importance of controlling and modulating systemic activity through appropriate immunosuppressive management ^4^.

In conclusion, accelerated atherosclerosis in AAV is a rare but widely recognized entity that requires a high clinical suspicion for early diagnosis and timely treatment, mainly due to the potentially life-threatening complications it entails. 
